# Electrospinning and Electrospraying: Emerging Techniques for Probiotic Stabilization and Application

**DOI:** 10.3390/polym15102402

**Published:** 2023-05-22

**Authors:** Kun Feng, Lulu Huangfu, Chuanduo Liu, Laura Bonfili, Qisen Xiang, Hong Wu, Yanhong Bai

**Affiliations:** 1 College of Food and Bioengineering, Zhengzhou University of Light Industry, Zhengzhou 450001, China; fengkun_89@163.com (K.F.);; 2 Key Laboratory of Cold Chain Food Processing and Safety Control, Ministry of Education, Zhengzhou University of Light Industry, Zhengzhou 450001, China; 3 Henan Key Laboratory of Cold Chain Food Quality and Safety Control, Zhengzhou 450001, China; 4 School of Biosciences and Veterinary Medicine, University of Camerino, 62032 Camerino, Italy; 5 School of Food Science and Engineering, South China University of Technology, Guangzhou 510640, China

**Keywords:** probiotic, electrospinning, dry electrospraying, wet electrospraying, encapsulation, immobilization, stabilization, colonic delivery

## Abstract

Probiotics are beneficial for human health. However, they are vulnerable to adverse effects during processing, storage, and passage through the gastrointestinal tract, thus reducing their viability. The exploration of strategies for probiotic stabilization is essential for application and function. Electrospinning and electrospraying, two electrohydrodynamic techniques with simple, mild, and versatile characteristics, have recently attracted increased interest for encapsulating and immobilizing probiotics to improve their survivability under harsh conditions and promoting high-viability delivery in the gastrointestinal tract. This review begins with a more detailed classification of electrospinning and electrospraying, especially dry electrospraying and wet electrospraying. The feasibility of electrospinning and electrospraying in the construction of probiotic carriers, as well as the efficacy of various formulations on the stabilization and colonic delivery of probiotics, are then discussed. Meanwhile, the current application of electrospun and electrosprayed probiotic formulations is introduced. Finally, the existing limitations and future opportunities for electrohydrodynamic techniques in probiotic stabilization are proposed and analyzed. This work comprehensively explains how electrospinning and electrospraying are used to stabilize probiotics, which may aid in their development in probiotic therapy and nutrition.

## 1. Introduction

Probiotics are living microorganisms that can benefit the health of the host when consumed in adequate amounts [1]. Probiotics belong to a few species, the most common of which are *Lactobacillus* spp. and *Bifidobacterium* spp. Other probiotics, such as bacteria and yeasts, are also available in the market [2,3]. However, the application of probiotics is often challenged by the adverse conditions during processing, storage, and gastrointestinal transit. Intensive research endeavors have been focused on addressing these limitations through adapting probiotic strains to stressors, screening acid- and bile-resistant strains, encapsulating/immobilizing probiotics, and so on [4,5,6,7]. Specifically, encapsulation and immobilization are two efficient approaches that aim to provide the probiotic cells with a microenvironment where the bacteria are protected from destructive conditions, maintaining their viability, as well as facilitating their stable delivery in the gastrointestinal tract. Although many techniques (e.g., freeze-drying, spray drying, and emulsification) have been applied to stabilize probiotics, some of their inherent drawbacks, including the use of extreme temperatures, the complexity of a multi-step procedure, and the use of organic solvents, would severely affect viability [8,9]. Therefore, exploring mild and simple encapsulation/immobilization strategies is crucial for preserving probiotic viability.

Electrospinning and electrospraying are electrohydrodynamic (EHD) techniques to produce nano/micro-scale fibers and particles that can be engineered for various applications [10]. In contrast to other techniques, electrospinning and electrospraying are versatile and straightforward processes that generally do not involve severe conditions (e.g., temperature, pressure). Recently, their superior performance in the stabilization and controlled release of sensitive components (e.g., essential oils, peptides, proteins) has gained great attention [11,12]. In particular, the stabilization of probiotics by electrospinning and electrospraying has been an emerging topic in the past few years. However, a systematic review of the progress of probiotic stabilization and applications using these two techniques is still not available. Furthermore, electrospraying can be separated into two different types based on the collector used, both of which are totally different in terms of probiotic encapsulation. Unfortunately, most studies have focused on the differences between electrospinning and electrospraying, and there is no detailed information about how electrospraying is classified and used in preserving probiotic viability.

Therefore, this paper aimed to provide an up-to-date and comprehensive overview of the stabilization and application of probiotics using two electrohydrodynamic techniques. Firstly, the principles of electrospinning and electrospraying are described. Specifically, a specific classification of electrospraying is emphasized to enable readers to differentiate and comprehend the application status of electrospraying for probiotic encapsulation. Subsequently, electrospun and electrosprayed probiotic stabilization systems developed by either encapsulation or immobilization and their capacities for enhancing probiotics’ survival under severe conditions are systematically discussed. In addition, the applications of probiotic formulations in several fields are summarized. Ultimately, the limitations of these two EHD techniques for probiotic stabilization and prospective research directions are proposed and discussed.

## 2. Electrospinning and Electrospraying

**Figure 1:**
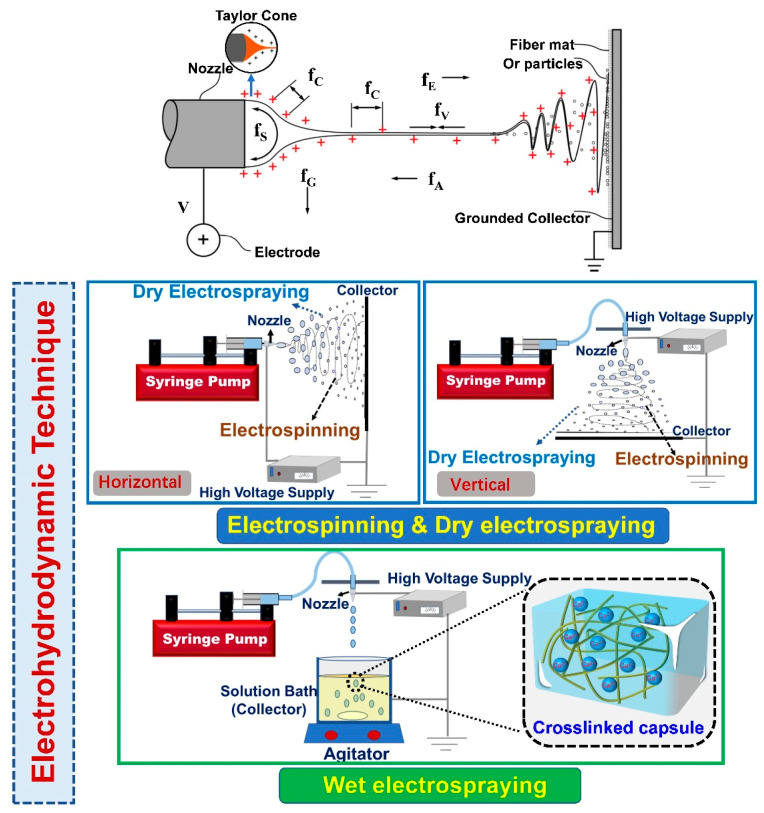
Forces governing the electrohydrodynamic process (f_A_, air drag; f_C_, coulombic force; f_E_, electric force; f_G_, gravitational force; f_S_, surface tension; f_V_, viscoelastic)-reprinted with permission from [15]. 2019, Elsevier; Schematic and classification of electrospinning and electrospraying.

**Figure 2:**
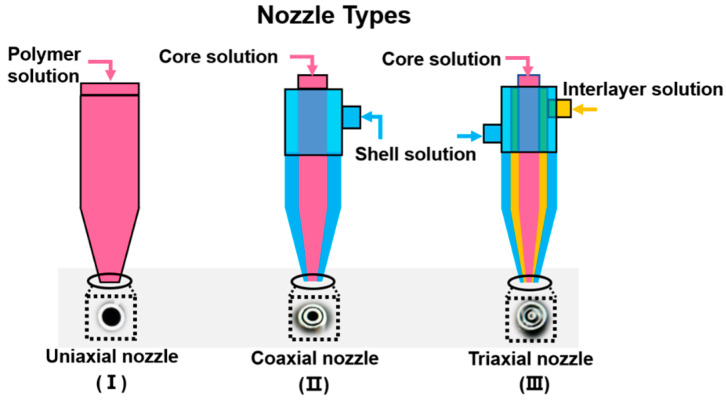
Typical nozzles used for electrospinning and electrospraying.

Although electrospinning and electrospraying are both classified as EHD techniques, they differ in several ways. Typically, the polymer solution is extruded from a needle using a syringe pump. Once the droplet emerges, a Taylor cone is formed on the needle tip owing to the balance between surface tension and electrical repulsion. Then, the jets will erupt from the cone when the repulsion forces overcome viscoelastic forces and surface tension; subsequently, the broken droplets will pull towards the collector. To date, the classification of electrospinning and electrospraying in most studies is crude. Indeed, electrospinning and electrospraying can be readily distinguished according to the collector used. As depicted in Figure 1, the collector device could be a solid plate (electrospinning and dry electrospraying) or a liquid bath (wet electrospraying). Electrospinning and electrospraying can be classified into three forms based on the nozzles used: uniaxial, coaxial, and triaxial nozzles, as illustrated in Figure 2. As for electrospinning, its detailed classifications (e.g., uniaxial electrospinning, coaxial electrospinning, emulsion electrospinning) have been widely reported in many existing reviews [13,14]. In contrast, the classification and influencing factors for electrospraying are still unclear and should be systematically discussed.

In most of the published reviews on electrospinning and electrospraying, the latter only refers to dry electrospraying [16,17]. It can be seen from Figure 1 that electrospinning and dry electrospraying have the same setup, collecting fibers (electrospinning) or particles (dry electrospraying) on the solid collector. The detailed influencing factors for electrospinning and dry electrospraying, including solution properties (e.g., conductivity, viscosity, surface tension) and technical parameters (e.g., voltage, distance, flow rate), have been comprehensively discussed in many previous reviews [12,18]. In fact, the difference in the properties of the polymeric solution is the main factor that determines the formation of fibers by electrospinning or solid particles by dry electrospraying. Generally, the increase of viscosity and reduction of surface tension facilitate the stabilization of the solution jet by chain entanglement, which thus inhibits the generation of microbeads and increases fiber diameter during electrospinning. On the contrary, polymer solutions with high surface tension and low viscosity tend to be electrosprayed into particles or beaded fibers. Polymer solutions that readily electrospray are typically those prepared from low-molecular-weight polymers with low concentrations [13]. Besides electrospinning and dry electrospraying, wet electrospraying has been another focus in probiotic stabilization. However, a specific description of wet electrospraying is still not available.

Wet electrospraying, which is totally distinct from both electrospinning and dry electrospraying, is commonly used to make hydrogel capsules by using the crosslinking agent (CaCl_2_) solely or together with other polyelectrolyte polymers (e.g., chitosan, whey protein isolate) as the collection solution [19,20]. Sodium alginate (SA) is the most used biopolymer in the area of wet electrospraying. The “egg-box” is formed by the interactions between the divalent cations and the carboxyl groups of the adjoining alginate [21]. As shown in Figure 1, wet electrospraying is typically performed by spinning the solution into the collection bath, where the particles are formed by the interactions of polymers and crosslink agents. To date, the influence of polymer concentration, crosslinking agent concentration, and processing parameters (i.e., voltage, distance, flow rate) on the properties of the produced capsules during wet electrospraying, especially capsule size, have been investigated in previous studies, whereas these research outcomes have not been systematically reviewed. Herein, these influences, according to previous studies, can be described into two parts: (1) polymer and crosslinking agent concentration and (2) wet electrospraying parameters. As for the polymer and crosslink agent, they are key factors that determine the formation of capsules. Generally, the unshaped and anomalous capsules tend to be formed at low concentrations due to the fact that the instantaneous crosslinking cannot be accomplished without a sufficient amount of SA and Ca^2+^ [22,23]. Moreover, coagulation is prone to occur under this condition. This phenomenon can be eliminated when the concentrations of SA and CaCl_2_ increase. Many studies have found that the increase in alginate concentration during certain ranges, for example, 0.5–1.5%, 1.5–2.25%, or 1–5% would result in an increased bead diameter [24,25,26]. However, further increase in SA concentration would either produce an anomalous bead morphology or even interrupt the electrospraying process by blocking the nozzle due to the increased viscosity of the polymer solutions. Regarding the crosslinking agent, increasing the CaCl_2_ concentration would accelerate the crosslinking process and reduce the time required for the formation of capsules. It has been demonstrated that single capsules started to form when the CaCl_2_ concentration increased and coagulation can be eliminated when the concentration increased to 0.5 M [27]. It was found that the size of capsules decreased with increasing CaCl_2_ concentration. A possible explanation is that the exchange of the crosslinking ions (Ca^2+^) from the crosslinking medium and water from SA droplets would tighten the gel network, resulting in smaller capsules. However, when sufficient Ca^2+^ fills up the cavities made up by the G-residues in the SA chain to a certain level, no further decrease could be achieved [28]. In addition, previous studies have indicated that the encapsulation efficiency of ingredients increased by increasing the SA and CaCl_2_ concentration in certain ranges. However, an excessive increase in concentration would inversely affect the encapsulation efficiency.

Unlike the above factors, research on the effect of electrospraying parameters (i.e., voltage, distance, and flow rate) on capsules has primarily focused on the capsule size. It is known that the strength of electric field can be calculated by dividing the voltage by the distance between the nozzle tip and the liquid collector. The variation in voltages and electrospraying distance directly affects the intensity of the electric field and, consequently, the diameter of the capsules formed. Voltage affects the formation of capsules by interfering with the surface tension between the droplet and the needle via ion migration of the SA solution [29]. Of note, three distinct modes (dropping mode, dripping mode, and cone-jet mode) can be found during the changes of voltage in the wet electrospraying process [25]. First, in the dropping mode (e.g., <10 kV) the electric field succumbs to the electric changes in surface tension, and large droplets are formed as a result of the interaction between gravity and surface tension. Then, an increase in voltage (e.g., >10 kV) leads to a non-stable surface area where a slight increase in voltage has a significant effect on the reduction of capsule size (dripping mode). When the voltage increases to a point, referred to as the critical voltage, a transition from dripping to jetting mode would result in the formation of fine droplets, and no further increase in applied voltage resulted in smaller capsules [30]. The cone-jet mode was featured in the formation of a stable Taylor cone, which is the most ideal mode for producing monodisperse capsules. These special models were also reported in other research [31,32]. Although distance also influences the size, a previous study revealed that the distance minimally affected the diameter of SA beads prepared at high voltages (>12 kV) [33]. As another crucial process parameter, flow rate also has an influence on the formation of capsules. It has been reported that the size of microbeads increased with the increase in flow rate under certain ranges because the solution flowed into the droplet before the detachment of SA droplet at the needle tip (e.g., 200 μL/min–600 μL/min). However, no further increase in capsule size was observed when the flow rate increased to a high value (e.g., 1000 μL/min) [27,29]. Therefore, it is necessary to fully study the influences of various parameters on the characteristics of hydrogel capsules during wet electrospraying.

## 3. Electrospinning for Probiotic Stabilization

Electrospinning is a practical technique to produce sub-micron or nano-scale fibers. The electrospun nanofibers exhibit diverse structural and functional characters, such as controllable diameters, porosity, high surface-to-volume ratio, and high encapsulation efficiency for bioactive compounds [15]. By virtue of these structural advantages, electrospinning has been widely used in the encapsulation and immobilization of bioactive compounds. From the encapsulation aspect, most research projects were performed using a small amount of active substance (e.g., curcumin, essential oil, quercetin, fish oil) [34,35,36,37] and active peptide/protein (e.g., lysozyme, insulin, calcitonin, lactoferrin, phycocyanin) [38,39,40,41,42]. It has been demonstrated that the bioactive ingredients embedded in electrospun fibers exhibited enhanced stability and bioavailability, achieving targeted delivery and sustained release [43,44,45]. With respect to immobilization, an electrospun fiber mat was primarily used as the matrix for immobilizing enzymes to improve their stability and reusability [46,47].

**Figure 3:**
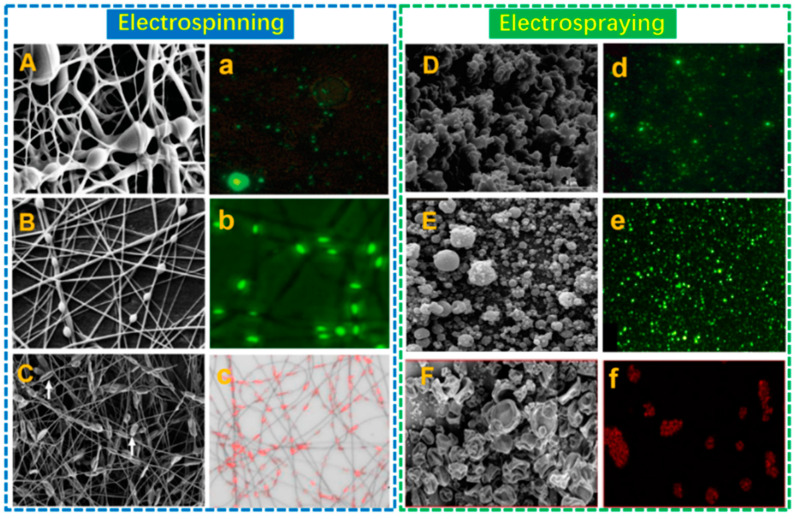
SEM and fluorescence images of probiotic fibers (**A**–**C**; **a**–**c**) and capsules (**D**–**F**; **d**–**f**) prepared by electrospinning and electrospraying-reprinted with permissions from [48,49,50,51,52,53]. 2011, American Chemical Society; 2020, Elsevier; 2019, Elsevier; 2012, Elsevier; 2019, Elsevier.

It is known that the stabilization of probiotics is crucial for their function. The great potential of electrospinning for probiotic stabilization has been confirmed and has gradually attracted increased attention in the past few years. Generally, the construction of probiotic formulations could be performed either by encapsulation or immobilization. Figure 3A–C) shows certain probiotic-loaded electrospun fibers and the matching fluorescence patterns of probiotic cells in these fibers (Figure 3a–c).

### 3.1. Electrospinning for Probiotic Encapsulation

#### 3.1.1. Feasibility of Electrospinning for Encapsulating Probiotics

Uniaxial electrospinning is a simple electrospinning method for encapsulating probiotics that involves mixing the polymer solution with the probiotics directly. In the beginning, the feasibility of encapsulating probiotics was verified by uniaxial electrospinning. In fact, the virus was initially employed as a model to evaluate the feasibility of electrospinning for encapsulating microorganisms. The M13 viruses were found to be encased in polyvinylpyrrolidone (PVP) fibers and maintained their capacity to infect [54]. Then, the capacity of electrospinning to encapsulate bacteria was further explored and compared to that of the virus. According to Salalha et al., bacteria are more resistant to the electrospinning process than the virus, due to the strong cell walls in the bacteria. In the area of bacteria encapsulation by electrospinning, the viability could be impacted by a variety of factors. For instance, smaller cells and Gram-positive bacteria were found to be more resistant to electrospinning [55,56]. Specifically, one study has explored and demonstrated that the Gram-negative bacterium seems to be more fragile than the Gram-positive bacterium due to the drastic change in the osmotic environment caused by the rapid evaporation of electrospinning solvent [57]. Probiotic viability is also linked to the hydrophobicity of the cells, while the hydrophilic probiotics exhibited a higher reduction in viability than the hydrophobic during the electrospinning process [58]. The reason behind this phenomenon is that the hydrophobic surface provides superior protection from the hydrophilic solution dispersed with the bacteria [59]. Aside from that, the high voltage used in the electrospinning process is thought to be hazardous to probiotics, even though it is required for nanofiber production. Herein, the effect of electrospinning voltage on bacteria viability has been examined. One study has reported that *Lactobacillus plantarum* (*L. plantarum*) incorporated in a polyethylene oxide (PEO) fiber mat at 15 kV had the greatest vitality (0.81 log reduction in viability of *L. plantarum*, in comparison to theoretical *L. plantarum* loading). Although the viability reduction of probiotics encapsulated in the fiber mat under voltage below or over 15 kV was slightly higher, the authors stated that the voltage used in the electrospinning process did not have any vital impact on the cell viability [53]. Similarly, in our previous study, no substantial viability reduction of probiotics during electrospinning was found in the voltage range of 10–16 kV [60]. Ceylan et al. also obtained an poly(vinyl alcohol) (PVA)-based electrospun fiber mat at 25 kV, in which the encapsulated probiotic also maintained high viability [61]. Another study demonstrated that no significant loss of viability for both *Lactobacilli* and *Bifidobacteria* was observed after the electrospinning process (24 kV) [62]. Therefore, previous studies all confirmed that the electrospinning approach has great potential for probiotics encapsulation. Generally, the electrospinning produces fibers with a nano/microscale diameter [63]. The encapsulation of probiotics in the fibers could lead to the formation of string, bead, or spindle fiber structures due to the excessive volume of probiotic cells. This occurs as a result of the bacteria being drawn into a sink-like flow during electrospinning, encasing them inside the fibers [55]. Moreover, it was found that the beads formed in the fibers were much smaller than the bacteria size [64]. To sum up, most studies reported that the probiotic cells were intact in the electrospun fibers, and the electrospinning process did not hurt the probiotic cells severely [49,56,65,66], indicating that electrospinning could be a promising approach for probiotic encapsulation.

#### 3.1.2. Modified Electrospinning Protocols for Probiotic Encapsulation

Previously, the encapsulation of probiotics was accomplished by uniaxial electrospinning with the synthetic polymers (e.g., PVA, PEO, PVP) as the wall material. Recently, many modified electrospinning protocols, including using binary electrospinning materials and coaxial electrospinning, were explored to encapsulate probiotics while evaluating their efficiency in improving the stability of encapsulated probiotics.

On account of the safety issue, natural biopolymers, such as proteins and polysaccharides, are considered as excellent encapsulation matrices, which have attracted great attention. However, the electro-spinnability of most polysaccharides and proteins is very poor due to their semicrystalline/crystalline property and intricate secondary/tertiary structures [67,68]. Their successful electrospinning generally requires the aid of synthetic polymers with good spinnability. Therefore, besides synthetic polymer electrospinning, binary systems made by combining synthetic polymers with other biopolymers (e.g., polysaccharides, protein) or additives (e.g., glycerol, lactose, mannitol, skim milk, prebiotic) were first investigated to encapsulate probiotics. As shown in Table 1, different binary systems used for probiotic encapsulation by uniaxial electrospinning were presented. The proper choice of binary system would be beneficial for the improvement of probiotic survivability under stress conditions. It has been reported that the viability of probiotics encapsulated in the synthetic polymer (PEO) fibers decreased by more than 2 log units after storage at 25 °C for 7 days, while the incorporation of disaccharides (i.e., sucrose, trehalose) in the fiber mat could decrease the viability loss during the electrospinning and storage process. This phenomenon was attributed to the amorphous disaccharides in the fibers and interactions between the disaccharides and probiotic cells [53]. In addition, prebiotics are indigestible food components that selectively stimulate the growth and/or activity of probiotics, thus providing beneficial effects for the host [69]. Several studies have proven that the addition of pre-biotic provided improved protection for probiotics [70,71]. However, to date, studies related to the use of prebiotics for probiotics encapsulation by electrospinning technique are very limited. Our team has attempted to establish a syn-biotic system for the first time by incorporating pre-biotic (fructo-oligosaccharide, FOS) into the PVA electrospinning system [49]. The results revealed that adding FOS to the probiotic carrier could significantly improve their proliferation and maintain high probiotic survival after heat treatment (45 °C, 60 °C, 70 °C) when compared to the free cells. Another study also found that the incorporation of prebiotics (three different inulin) in PVA/SA fiber mat could provide protection for the probiotics and improve their survivability during storage at −18 °C, 4 °C, and 25 °C, but the protection effect varied according to the polymerization degree, concentration, and dissolution of the prebiotic used [72]. However, most binary systems still consist of synthetic polymers, which do not fit in with the improved food safety awareness of consumers. Herein, in order to ensure oral safety, more work should be put into developing food-grade probiotic carriers that do not use synthetic polymers or harsh solvents in the electrospinning process. By doing this, the solution properties, such as viscosity, conductivity, and surface tension, should be carefully considered and controlled. For example, Liu et al. used two edible polysaccharides (pectin and pullulan) to create food-grade ultrafine fibers for encapsulating *Lactobacillus rhamnosus* (*L. rhamnosus*) GG [73]. The inclusion of pullulan could reduce the surface tension and electric conductivity of pectin solution, probably by restricting the mobility of sodium ions of pectin in the solution. Therefore, pullulan could be substituted for other synthetic polymers (e.g., PVA, PEO) in the electrospinning process to make pectin fibers. In addition, gum Arabic was also used to fabricate a probiotic fiber mat with pullulan with an optimized mass ratio of 20:80 [65]. The survival rate of *Lactobacillus* encapsulated in Arabic/pullulan electrospun nanofibers was higher than that of freeze-dried carriers (85.38~97.83% vs. 80.92~89.84%), and the viability was maintained during 28 days of storage at 4°C. In another work, the co-electrospinning of corn starch and sodium alginate was investigated to encapsulate several probiotic strains [74]. The probiotics were well protected by the fiber mat, which retained more than 95% of their viability after 20 days of storage in the yogurt. Therefore, based on current knowledge of probiotic encapsulation by electrospinning, it is essential to further explore more food-grade materials or compositions for developing efficient probiotic carriers, boosting their application in the functional food industry.

**Figure 4:**
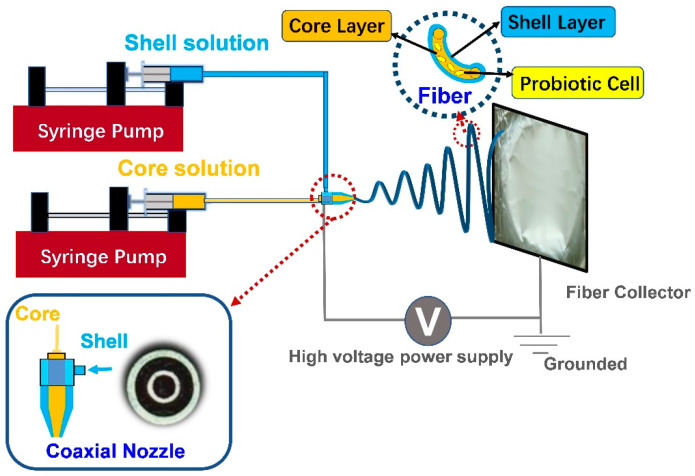
Double-layered encapsulation of probiotics by coaxial electrospinning-reprinted with permission from [49]. 2020, Elsevier.

In addition to uniaxial electrospinning, another important strategy for probiotic encapsulation is coaxial electrospinning. In coaxial electrospinning, two concentrically arranged spinnerets are connected to two reservoirs carrying different spinning fluids, as shown in Figure 4. It is well understood that the encapsulation of probiotics is to provide a protective layer to improve their stability under harsh conditions and reduce viability loss. Coaxial electrospinning, in this sense, is a gentle and simple approach to developing composited fiber with a core/shell structure. The embedding of probiotic cells into the core could provide an extra protective layer (shell) than the uniaxial electrospun fiber. A previous study indicated that the Bifidobacterium animalis (*B. animalis*) Bb12 incorporated into the coaxial poly(vinyl alcohol) (PVOH) fibers remained at a higher viability for 140 days at 4 °C, which was better than the uniaxial fiber mat [91]. In addition, another benefit of coaxial electrospinning is the ability to use a probiotic-friendly fluid medium as the core material, which is not required to be spinnable, since it is entrained by the shell polymer. For instance, Lancuški and coworkers developed a starch-formate composite fiber by coaxial electrospinning using glycerol as a dispersion medium in the core [92]. The successful encapsulation of Lactobacillus paracasei (*L. paracasei*) within the compound fibers could maintain viability at 4 °C and 25 °C for three weeks, providing a novel route for probiotic stabilization.

#### 3.1.3. Potential of Electrospun Fiber Mat for Delivering Probiotics to the Colon

In the past, research has mainly focused on evaluating the stability of encapsulated probiotics in the fiber mat during the encapsulation and storage process. Nevertheless, probiotics are required to survive harsh upper gastrointestinal conditions in order to finally colonize and function in the colon [93]. Based on previous studies, three strategies related to selecting electrospinning materials, constructing a multi-layered structure, and improving carrier hydrophobicity have been applied to improve the survivability of probiotics under in vitro digestion conditions.

For the oral delivery of probiotics, encapsulation could provide probiotics with a protective layer to separate them from the harsh digestive conditions. Herein, polymers that were used to produce the delivery vehicles should be able to resist the destruction factors in the gastrointestinal tract. Polysaccharides, such as chitosan, pectin, and alginate, commonly used for the construction of colonic delivery systems, were also applied to promote the stable delivery of probiotics to the colon [94]. Mojaveri et al. stated that chitosan (CS) is very stable in the acidic environment of the stomach and found that the encapsulation of B. animalis in the PVA/CS fiber mat could remarkably increase their survivability under simulated gastric fluids (SGF) and simulated intestinal fluids (SIF) when compared to the PVA fiber mat [81]. Similarly, other electrospun probiotic carriers made from starch/SA, PVA/SA/inulin, or PVA/Gum Arabic have been shown to have high probiotic survival after exposure to the simulated digestion medium [62,72,83]. In addition, the silk fibroin (SF) was also used to produce a *L. plantarum*-loaded PVA/SF nanofiber mat. The encapsulated probiotics exhibited obviously increased survival rates, after being treated with SGF for 2 h. This phenomenon was explained by the fact that the H^+^ entering the fiber mat could be neutralized by the free acid amino acid in the PVA/SF fibers [85]. Apart from that, prebiotics are resistant to low acidity and digestion in the upper gastrointestinal system [95]. As a result, their utilization in the construction of efficient delivery systems for probiotics has recently attracted increased interest. Duman and Karadag found that probiotics in the fibers enriched with prebiotic (inulin) showed higher viability against simulated gastrointestinal conditions [72]. Therefore, the syn-biotic encapsulation system seems to be a promising strategy for probiotic stabilization, and the internal mechanism should be further investigated.

Another efficient strategy is to construct a multi-layered protective structure for probiotics. In this regard, coaxial electrospinning and tri-axial electrospinning are two simple and flexible approaches to fabrication of double-layered and tri-layered fibers. However, their usage in probiotic encapsulation is still in its early stage. As shown in Figure 4, our group has developed a core/shell carrier through a one-step coaxial electrospinning with alginate as the shell material [69]. The core/shell fibers improved probiotics’ tolerance to the simulated gastric fluid and small intestine fluid, and no significant viability loss was found for the encapsulated probiotics after exposure to the above fluids. Moreover, compared to cells encapsulated in a uniaxial fiber mat, the encapsulated cells demonstrated greater thermal stability under heat-moisture treatment and exhibited a lower reduction in viability. Meanwhile, Yu et al. fabricated a fiber mat with polylactic acid (PLA) and FOS as the shell by coaxial electrospinning [96]. They reported that PLA is well-known for its good acid resistance, which could keep the fiber mat stable in the gastric acid environment while gradually releasing the lactic acid bacteria in the simulated intestinal fluid through the slow dissolution of the PLA shell. The viable count of bacteria was 8 × 10^3^ CFU/mg, and more than 72% of bacteria survived after 2 h of treatment with the simulated intestinal solution. In a recent study, a core-shell fiber mat has been developed to encapsulate probiotic Lactobacillus rhamnosus 1.0320, in which a pH-sensitive polymer, Eudragit S100 (ES100), was used as the shell to resist the destruction due to harsh acid conditions. It was found that an 84.3% survival rate of the encapsulated probiotic cells could be obtained after the treatment of simulated gastrointestinal solutions [97]. It is known that carriers with more compact and complex structures are more conducive to probiotic viability. Tri-axial electrospinning has been reported for colon-targeted drug delivery. Even though it has not been used for probiotic encapsulation, it will be a focus of future studies. Therefore, exploring specific electrospinning strategies to supply probiotic extra protective layers is a promising way to achieve high-viability probiotic delivery to the colon.

**Figure 5:**
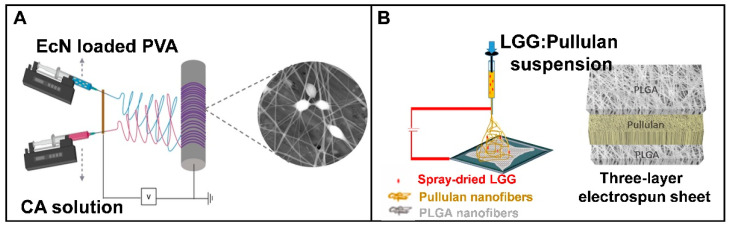
Modified electrospinning protocols for preparing hydrophobic probiotic carriers: (**A**) dual-nozzle electrospinning (PVA: poly(vinyl alcohol), CA: cellulose acetate)-reprinted with permissions from [98]. 2021, Elsevier; (**B**) Layer-by-layer electrospinning (LGG: Lactobacillus rhamnosus GG)-reprinted with permissions from [99]. 2022, Elsevier.

So far, most of the emphasis has been concentrated on encapsulating probiotics in an electrospun fiber mat with either a mono-layer or core-shell structure, most of which were made up of various hydrophilic polymers. One main reason is that the electrospinning of hydrophilic polymers could avoid the use of harsh solvents, which would not affect viability. Despite comparable/improved survival and viability when compared to other conventional carriers, the intrinsic hydrophilic feature of the probiotic carrier will challenge the delivery efficiency of probiotic cells to the colon site. Herein, polymers that are weakly soluble in water have drawn increased interest due to their excellent water stability. By considering this issue, researchers have attempted to improve the hydrophobic property of carriers or develop a unique fiber structure by modifying the previously used electrospinning protocol. Çanga and Dudak used an angled dual-nozzle electrospinning method to prepare PVA/cellulose acetate (CA) fiber mats to encapsulate Escherichia coli Nissle 1917 (EcN) [98]. The probiotic-incorporated PVA fibers and the CA fibers were electrospun from different nozzles to avoid the contact of cells with the solvent of CA (Figure 5A). The results demonstrated that probiotic cells were encased in the PVA/PVA and PVA/CA fibers without significant loss of viability during the whole process. Furthermore, the probiotic cells in the PVA/CA-EcN fibers were more resistant to stomach simulation than those within the PVA/PVA-EcN fiber mats, with a 2.0 log CFU/mL drop in cell viability after 2 h of SGF treatment. In addition, another study was performed to generate a sandwich structure carrier for L. rhamnosus GG utilizing a layer-by-layer electrospinning strategy, as presented in Figure 5B, in which an edible water-soluble polysaccharide (pullulan) was employed for bacteria encapsulation, and hydrophobic polylactic-co-glycolic acid (PLGA) polymer was used as the upper and bottom layer to protect the inner layer during the storage and gastrointestinal tract transition [99]. In vivo study showed that Lactobacillus rhamnosus GG (LGG) cells could be delivered by the multilayer carrier survived intestinal transit and were recovered from all segments of the intestine. These findings pave a new route for the development of probiotic carriers with improved capabilities for protecting the loaded probiotic cells.

### 3.2. Electrospinning for Probiotics Immobilization

The stabilization of probiotics is meant to maintain viability during processing and the passage through the gastrointestinal tract, and to realize a stable delivery of probiotics by means of different probiotic carriers. Electrospun nanofiber mats have loosely connected 3D porous structures with high porosity and surface area. This unique structure can perfectly mimic the extracellular matrix’s natural structure for cell adhesion and proliferation [100]. Hence, aside from probiotic encapsulation, an electrospun nanofiber membrane is another important approach to probiotics stabilization. The immobilization of probiotics on the electrospun fiber mat is usually achieved by immersing the fiber mat into a probiotic suspension to absorb bacterial cells. Indeed, bacterial immobilization on the electrospun fiber mat was initially applied in wastewater treatment [101,102]. In recent years, the feasibility of the immobilization of probiotics on the fiber mat has been assessed. Hu et al. used an electrospun cellulose acetate fiber mat to immobilize *L. plantarum* in order to study the formation of the probiotic film [103]. The *L. plantarum* biofilms-loaded membrane was employed as reusable starter culture in the milk fermentation, and its effect on the fermentation properties was investigated. They found that *L. plantarum* biofilms on the fiber matrix exhibited excellent gastrointestinal resistance compared to that of the planktonic bacteria. The fermented milk produced with the probiotic biofilm-integrated nanofiber mats as the starter exhibited a shorter fermentation time and higher survival of probiotics during shelf life. Our recent study also immobilized probiotic cells using the ethyl cellulose (EC) fiber mat as support and investigated the factors that influence the immobilization and biofilm formations of probiotics. It is known that cell growth and proliferation are both influenced by the roughness and hydrophobicity of the immobilization matrix [104,105]. Interestingly, it was confirmed that the hydrophobicity and surface roughness of the electrospun EC fiber mat were beneficial for the immobilization of *L. plantarum*. Moreover, it was found that the luxS gene was one key factor in biofilm formation, because its relative expression level was 8.7-fold higher than that of the planktonic cells. The biofilm on the fiber mat could improve the thermal stability of immobilized probiotic cells, as well as survivability in the simulated gastrointestinal conditions [106]. In another work, the effect of an electrospun PVP fiber mat on the metabolic activity of immobilized probiotics (*L. acidophilus*, *L. acidophilus*) was investigated [107]. The enzyme activity and production of DL-lactic acid were used to assess the metabolic activity of immobilized probiotics. There was an increase in viable probiotic cells up to 7.4 × 10^8^ CFU/mL in the presence of PVP, as well as an accumulation of DL-lactic acid. They hypothesized that the degree of cell aggregation is determined by the proliferation of microorganisms in the presence of PVP nanofiber mats, indirectly affecting the synthesis of secondary metabolites. Another method for the immobilization of probiotics on the fiber mat was described by Jayani et al., who immobilized the *L. acidophilus* onto the bacterial cellulose nanofibers through the absorption-incubation method [108]. The immobilized probiotic survived for up to 24 days at room temperature with a survival rate of 71.1%. The thermal experiment indicated that this electrospun fiber mat could be used heat-processed foods due to their nanostructure and superior stability up to 180 °C. Grzywaczyk et al. also created a new route for immobilizing probiotics, which involved incorporating the electrosprayed probiotic (*L. plantarum*) particles into a sandwich-structure made of pre-and post-electrospun polystyrene fiber mat [109]. A significant reduction in the destructive effect of UV radiation and heat treatment (50 °C, 24 h) was observed when the bacteria were immobilized in the composite, as compared to free cells and cells in alginate only. In addition, irrespective of the probiotic immobilization, the probiotic-iron (III) oxide nanoparticle complex was biosynthesized and immobilized on the electrospun PVA/gum arabic (GA)/polycaprolactone (PCL) fiber mat to produce a multifunctional coating material. The integration of Fe_2_O_3_ nanoparticles, prebiotic GA, and probiotic Lactobacillus could promote antibacterial, antifungal and antibiofilm activity due to the synergistic effect [110]. Altogether, both studies proved the feasibility of immobilizing probiotics using the electrospun fiber mat as the loading medium to protect them from harsh circumstances, paving an alternative method for the design of novel delivery systems for probiotics.

## 4. Electrospraying for Probiotic Stabilization

**Figure 6:**
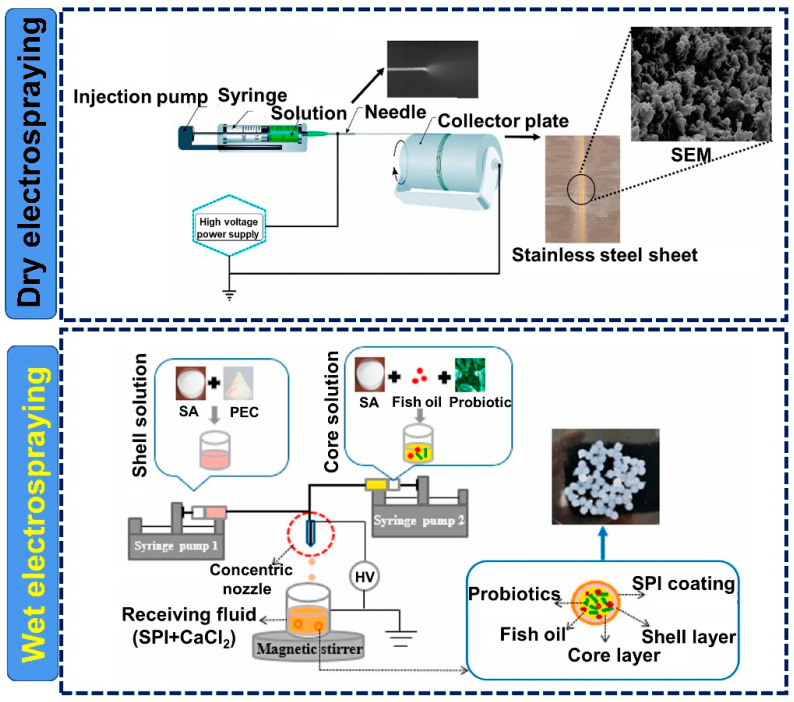
Encapsulation of probiotics by two electrospraying techniques (SA: sodium alginate; PEC: pectin; SPI: soy protein isolate)-reprinted with permissions from [20,111]. 2021, Elsevier; 2021, Elsevier.

Electrospraying, another type of EHD, has been recognized as an emerging technique for probiotic stabilization. Noteworthily, as distinct to electrospinning, studies in this area are only conducted by encapsulating probiotics into the particles (Figure 3D–F), either by dry electrospraying or wet electrospraying (Figure 6), as described in Table 2. No study has used electrosprayed formulations as the matrix to immobilize probiotics.

### 4.1. Dry Electrospraying for Probiotic Encapsulation

As described in Section 2, dry electrospinning has the same collector as electrospinning, while the difference is that the formulation fabricated by dry electrospraying consists of solid particles. Recently, the workability of dry electrospraying has been investigated. Its effectiveness in encapsulating and preserving various probiotic strains has been compared to that of other traditional techniques (i.e., freeze drying). For example, Moayyedi et al. examined the feasibility of microencapsulating *L. rhamnosus* ATCC 7469 in whey protein isolate, whey protein isolate + inulin or whey protein isolate + inulin + Persian gum matrixes, using electrospraying, spray drying, and freeze-drying methods [115]. They stated that electrospraying was shown to be more harmful to the sensitivity of *L. rhamnosus* ATCC 7469 cells than the other two techniques. Moreover, the electrosprayed probiotics suffered a higher viability loss during storage and the treatment of simulated digestive media when compared to the probiotics encapsulated by freeze drying and spray drying. The injury mechanism of electrospraying on probiotics was not fully understood and the authors speculated that the possible reason was the high voltage used in the electrospraying. However, other similar studies reported contradictory results. López-Rubio et al. fabricated whey protein concentrate/pullulan-based capsules for *Bifidobacterial animalis* Bb12 encapsulation by electrospraying [51]. Loss of viability was not observed during the electrospraying encapsulation. Moreover, electrosprayed encapsulation could prolong the survival of probiotics during storage at 4 °C and 20 °C and at various relative humidity conditions compared to freeze-dried probiotics. Another study also demonstrated that the survival rate of *L. casei* with electrospraying was close to 99.4% and that for freeze drying it was determined to be 84.2%. The difference can be attributed to the lower stress during probiotic encapsulation by electrospraying, in contrast to freeze drying [52]. Therefore, dry electrospinning is confirmed to successfully encapsulate probiotics and guarantee their viability during dry electrospraying.

In addition to uniaxial electrospraying, coaxial electrospraying was applied for the first time to encapsulate probiotics in a composite structure with gelatin and whey protein concentrate (WPC) as the shell and core materials, respectively [113]. Unfortunately, results showed that the composite structure could not protect *L. plantarum* from thermal stress, high humidity, or in vitro digestion, whereas capsules created by uniaxial electrospraying presented a higher survival rate. The authors presumed that the acetic acid used as the shell solvent seemed to irreversibly damage cell viability, making the cells more susceptible to harsh conditions and hastening their decay during storage. The low pH and osmotic shock in the electrospraying process were believed to cause damage to probiotic cells. Moreover, the residual acetic acid in the carrier would also probably impact probiotic survival. Considering this, a modified coaxial electrospraying strategy was created to construct a core-shell microcapsule for probiotic *B. animalis*. Of note, several ingredients (concentrated Bifido, Bifido-maltodextrin, and Bifido-glycerol) were supplemented as the core components and their effect on the probiotic stability was assessed when ethanol or acetone was selected as the solvent for the shell material (ethyl-cellulose) [53]. It was found that the addition of additives (maltodextrin or glycerol) in the core had a protective effect on cell survival. Interestingly, a unique interface that could separate the capsule’s core (Bifido cells and glycerol) from the shell was clearly visible in the cross-section observation of the core-shell capsule. In this case, it was speculated that the faster evaporation of shell solvent (acetone) and the subsequent solidification of the shell material prevented the mixing of core and shell materials. Therefore, this study suggested that the probiotics encapsulated in the core/shell capsules maintained viability after electrospraying, even though the shell layer was prepared using a solvent that typically reduces cell viability.

In the field of probiotic encapsulation by dry electrospraying, WPI and WPC are the most commonly used wall materials to encapsulate probiotics. Even though probiotics encapsulated in electrosprayed WPC capsules have been found to survive in simulated digestion conditions, protective capability still needs to improve [112]. The water solubility of whey protein will break up the structure of the capsules, compromising probiotic survival in the gastrointestinal tract. To address this issue, a food grade cross-linker, transglutaminase enzyme (TG), was applied to treat whey proteins (WPC and WPI) prior to dry electrospraying. Interestingly, capsules fabricated with TG-treated WPC and WPI were insoluble in water and highly resistant to SGF and SIF, resulting in the highest survival of *L. casei* following sequential incubation in simulated gastrointestinal conditions [52]. Therefore, further research should be carried out to identify appropriate dry electrospraying procedures and protective polymers/additives for efficient encapsulation and protection of probiotics.

### 4.2. Wet Electrospraying for Probiotic Encapsulation

In contrast to dry electrospraying, wet electrospraying is more commonly used in the preparation of probiotic carriers. For wet electrospraying, probiotic suspension, typically mixed with polysaccharide solution, is generally extruded into the gelling solution (i.e., CaCl_2_) to form hydrogel capsules. The most commonly employed polysaccharides are sodium alginate (SA) and pectin due to their bioavailability, non-toxic nature, biocompatibility, and ease of preparation as ionotropic gelation. The capability for high encapsulation of viable cells (almost 98%) of this technique has been confirmed previously [120]. In comparison to dry electrospraying, the majority of wet electrospraying studies focused on constructing effective encapsulation vehicles to improve the survival of probiotics when exposure to harsh gastrointestinal conditions. For example, Coghetto et al. encapsulated the *L. plantarum* in SA or pectin-SA matrix and investigated their protective capability for probiotics under simulated gastric acid and intestinal fluids [118]. The electrosprayed microcapsules were able to prolong *L. plantarum* BL 011 survival when exposed to simulated gastrointestinal fluids and during 21 days of storage at 4 °C. Likewise, the same research group also developed an SA-based microcapsule for *L. plantarum* [119]. The microcapsule exhibited significant cell survival under gastrointestinal fluid conditions, as well as during long-term storage (6 months) at 25 °C, retaining cell viability within the range required for food addition.

Despite this, many studies have found that the alginate component alone was unable to improve cell viability in the stomach environment effectively. Moreover, uncoated capsules are also susceptible to chelating agents (phosphate and citrate), resulting in a faster release of core materials. In this case, the combining of SA with other polymers, either by blending or coating, is one possible solution to the constraint. For instance, a core-shell microcapsule was prepared by coating the SA microcapsules with zein, an edible hydrophobic polymer that may aid in retarding SA microcapsule degradation in the small intestine, thus increasing the availability of probiotics incorporated into the core matrix [117]. Results of this study demonstrated that the encapsulated *L. acidophilus* suffered only a 1-log reduction after 2 h of incubation in SGF at pH 1.2 with pepsin, but the number of non-encapsulated bacteria was reduced by nearly 5-log cycles. Another strategy was to cover the SA microcapsule with a polycation to generate a complex structure based on the strong electrostatic attractive forces between acid and amine residues, which helped to further stabilize the microcapsule at a low pH condition. However, Zaeim, et al. demonstrated that the chitosan-coated SA microcapsule protected bacteria more effectively at a low pH environment when compared to the chitosan/SA blending group [120]. The fact is that blending chitosan with SA to produce microcapsules brings chitosan molecules close to probiotic cells, compromising their viability and reducing their resistance to a low pH environment. In contrast to CS, another work demonstrated that the presence of resistant starch inside the SA matrix provided a comparable level of protection to the chitosan-coated SA microcapsule during the digesting process [121]. These results were explained by the fact that the presence of resistant starch in the SA matrix may serve as a source of energy and carbon for probiotics. Unfortunately, the starch/SA microcapsule was less retentive on the mucosa due to the non-ionic property of starch. The chitosan-coating SA microcapsule, however, possessed a better mucoadhesive property. Thereby, the proper choice of materials for wet electrospraying is critical for developing effective stabilization systems for probiotic survival and colonizing.

In addition to the formulations made only of biopolymers, a syn-biotic system fabricated by wet electrospinning has also aroused increased attention in recent years. Probiotic vehicles enriched with different prebiotics were developed and their efficiency for probiotic stabilization under various conditions were evaluated. For example, a double-layered Ca-SA/chitosan microcapsule for *L. plantarum* and *Bifidobacterium lactis* was fabricated, in which either inulin or resistant starch was co-encapsulated [121]. Interestingly, microcapsules containing resistant starch performed better in terms of preserving probiotic viability under simulated gastrointestinal conditions than the inulin group. The authors illustrated that the starch had small granules, which provided a larger surface for bacteria adhesion, probably supporting the recovery and growth of probiotics after the transition through the upper gastrointestinal tract. This result agreed with other reported inulin-incorporated syn-biotic systems [123,124,125]. In addition, our group has successfully constructed a colon-targeted system with a multilayer structure by integrating coaxial electro-spraying with the coating method, as displayed in Figure 6. In the protocol, SA/pectin and SA/probiotics/fish oil were served as the shell and core solutions in the coaxial electrospraying process, respectively, and soybean protein isolate was employed as the coating material stored in the gelling bath [20]. According to the latest definition, polyunsaturated fatty acids were also classified as prebiotic [126]. Omega-3 fatty acids have been reported to promote probiotic growth and to help them to adhere to the intestinal wall [127]. In this regard, fish oil, which is rich in omega-3 fatty acids, was used as the core substance. The workability of the fish oil enriched symbiotic system on the stability of probiotics under various severe conditions was investigated. It was found that the addition of fish oil into the encapsulation system dramatically improved the survival of probiotic cells under the designed stress environments (heat treatment, storage, and freeze-drying), revealing that the prepared syn-biotic system is a promising vehicle for colonic delivery of probiotics.

## 5. Applications of Electrospun/Electrosprayed Probiotic Formulations

Based on the overview of the electrodynamic encapsulation and immobilization of probiotics, most methods focused on creating probiotic carriers and evaluating their protective capabilities under various conditions. Furthermore, the application potential of electrospun or electrosprayed probiotic formulations in some fields, such as tissue healing, food preservation, and yogurt/fruit juice, has also been evaluated.

### 5.1. Tissue Healing

**Figure 7:**
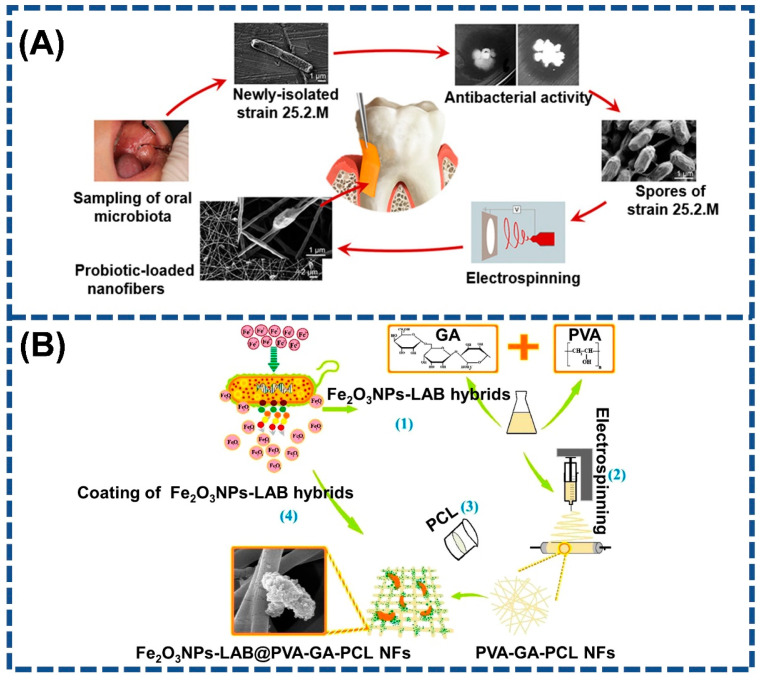
Application of electrospun probiotic fiber mat in the tissue healing: (**A**) periodontal disease-reprinted with permission from [79]. 2018, American Chemical Society; (**B**) Wound healing (GA: gum Arabic; PVA: poly(vinyl alcohol); PCL: polycaprolactone)-reprinted with permission from [110]. 2022, Elsevier.

In comparison to traditional treatment, probiotic recolonization is now recognized as a novel strategy for treating periodontal disease. However, this approach has disadvantages, including a shortage of powerful probiotics isolated from the human oral microbiota and a less efficient delivery system for prolonged probiotic retention in the periodontal pocket. Given this, Zupančič et al. isolated a probiotic strain from the oral microbiota [79]. Then, electrospinning was used to construct a delivery system for this probiotic. By means of this electrospun carrier, the viability of probiotics was maintained during electrospinning and 12 months of storage, and the delayed release of probiotics contributed to the improved antimicrobial activity against periodontal pathogens (Figure 7A). Additionally, the good performance of probiotic-incorporated electrospun fiber mat for burn healing was confirmed in another study [80]. In BALB/c mice with a second-degree contact burn, the probiotic (*Enterococcus mundtii*) functionalized scaffold could inhibit harmful bacteria while also accelerating epithelialization, collagen deposition, and hair follicle formation. Another new approach potentially used for wound healing was performed using the probiotic-metal oxide nanoparticle coated electrospun fiber mat. For example, lactic acid bacteria (LAB) were employed to synthesize green metal nanoparticles owing to their bio-reduction sites. As reported by the same research group, the electrospun fiber mat was first prepared using the gum Arabic, PVA, and PCL. Two different metal nanoparticles (NP), LAB-ZnO NP and LAB-Fe_2_O_3_ NP, were synthesized utilizing two different *Lactobacillus* strains (i.e., *L. plantarum* and *L. acidophilus*). The LAB-ZnO@GA/PVA/PCL fiber mat and LAB-Fe_2_O_3_ NP@GA/PVA/PCL fiber mat obtained demonstrated improved antibacterial and antibiofilm efficiency, implying therapeutic potential for wound healing application (Figure 7B) [110,128].

### 5.2. Food Preservation

Instead of conventional preservation strategies, biodegradable polymers or natural bioactive compounds are becoming more important in food research. Lactic acid bacteria are able to create an acidic environment that inhibits the proliferation of certain harmful bacteria. The critical role of *L. rhamnosus* in prolonging the shelf life of particular foods has been proven [129]. Therefore, the researchers developed an *L. rhamnosus* encapsulated electrospun fiber mat and systematically evaluated its role in preserving fish fillets [76]. The results indicated that using the probiotic-loaded fiber mat could delay the growth of total mesophilic aerobic and psychrophilic bacteria in the fish fillets by up to 38%, which could help with fish fillets preservation. Meanwhile, the same research group investigated the stability of polyunsaturated fatty acids (PUFA) and monounsaturated fatty acids (MUFA) in fish fillets using a PVA/SA fiber mat encapsulated with or without probiotic *L. rhamnosus* (LR or PS) [78]. Results showed that fish fillets coated with both the LR and PS could restrict changes in the PUFA and MUFA when compared to the uncoated group. The loading of *L. rhamnosus* to PS could particularly provide a better higher atherogenic and thrombogenic index during the storage. Furthermore, it was confirmed in another study that coating the fillets with the *L. rhamnosus* incorporated PVA fiber mat could significantly increase the inhibition of free radicals in the fillet samples compared to fish fillet samples from the control group [61].

### 5.3. Food Processing

The electrospun cellulose acetate fiber mat was supplied as the basis for the formation of *L. plantarum* biofilm, and the *L. plantarum* biofilm-integrated fiber mat was further used as the starter culture in the milk fermentation [103]. This fiber mat was found to possess good reusability and reliability in producing fermented milk. Furthermore, the fermented milk had a shortened fermentation period and a higher probiotic survival during shelf life. In another study, kefir was used as the food model to examine the survival of probiotics encapsulated in the electrospun PVA/SA fiber mat [82]. The nanoencapsulation of *L. paracasei* in the fiber mat could improve its viability in kefir, according to the in-situ viability test. There was no significant change in the flow behavior and viscoelastic nature of kefir inoculated with the encapsulated probiotics. Regarding the application of electrosprayed microcapsules, in one study, Ca-SA microcapsules encapsulated with *L. plantarum* were added to the orange juice. The survival of the probiotic cells during storage and the sensory acceptance of the juice were investigated [119]. The encapsulated probiotics exhibited significant resistance to orange juice, and only 2.4 log CFU/mL of viability loss was determined. The orange juice supplemented with this probiotic microcapsule had a good acceptance rate (>88%). Overall, the probiotic formulations prepared by these electrohydrodynamic techniques could be an alternative for producing probiotic-enriched functional foods.

## 6. Conclusions and Perspectives

Probiotics are susceptible to harsh food processing and gastrointestinal digestion, restricting their application in the food industry, as well as their bioavailability in the body. Electrospinning and electrospraying, known as electrohydrodynamic techniques, have attracted much interest as an emerging strategy for stabilizing probiotics in recent years. However, they differ in many aspects when they are used to construct probiotic carriers. As for electrospinning, it could stabilize probiotics by either encapsulating them in the fibers or immobilizing them on the fiber mat. Generally, synthesis polymers are the main materials used for the preparation of probiotic carriers due to their good electro-spinnability. The formation of fibers is determined by the properties of polymers and electrospinning parameters. With regards to electrospraying, dry electrospraying and wet electrospraying are used to stabilize probiotics by encapsulating probiotics in the capsules. The differences between them lie in the materials, production process, and capsule forms. Dry electrospraying has the same setup as electrospinning (Figure 1). The capsules could be prepared with either synthetic polymers or natural polymers. While wet electrospraying is generally performed using natural polymers to produce hydrogel capsules, of note, crosslinking agents are the dominant factor in the formation of this type of capsule. Unlike electrospinning and dry electrospraying, some probiotic cells could not be incorporated into the hydrogel capsules during wet electrospraying due to the slow gelation process, which would result in a lower encapsulation efficiency. As described in the sections above, the electrohydrodynamic encapsulation and immobilization of probiotics could provide effective protection and maintain their viability under a variety of stress conditions. However, the usage of these techniques for stabilization and targeted delivery of probiotics is still in its early stages, and research on their downstream applications in the food industry is scarce. Consequently, the following aspects are current issues and limitations in this area that should be considered in the future.

Probiotics are living microorganisms that can confer health advantages, as indicated in the definition. The exploration of stabilization techniques assures that probiotics are stable and have high viability during food processing and administration in the body. In this sense, the safety of materials for probiotics is vital for their use in the food industry. Currently, the production of most probiotic carriers by an electrohydrodynamic technique, especially electrospinning, has required the utilization of synthetic polymers (e.g., PVA, PEO, PCL) due to their good electro-spinnability. However, the oral safety of these synthetic polymers is still controversial. Fortunately, the viscosity and conductivity of polymer solution are key factors that determined successful electrospinning and electrospraying. Even though most biopolymers (e.g., polysaccharides, proteins) have poor electro-spinnability, a reasonable combination of these biopolymers could modulate the properties of the mixed solution and promote the generation of electrospun fibers or electrosprayed microcapsules. Thereby, further efforts should be taken to explore more food-grade biopolymers to prepare efficient vehicles for probiotic stabilization. On the other hand, the addition of some functional additives (e.g., prebiotic, protectant) could help to improve the survival of probiotics under harsh conditions. However, insights into the protective mechanism developed through this strategy need to be more deeply investigated.

In terms of the carrier structure, probiotics encapsulated in the multilayered fibers or microcapsules generally exhibit improved viability under severe conditions due to the extra protective layer. Nevertheless, in the area of electrospinning, previous studies have mainly focused on the verification of electrospinning on the encapsulation and immobilization of probiotics, which is commonly performed by uniaxial electrospinning. Therefore, the modification of electrospinning configurations, such as coaxial electrospinning, triaxial electrospinning, multi-nozzle electrospinning, and layer-by-layer electrospinning, should be carried out to develop multilayered systems for probiotics. In addition, most previous studies have focused on building probiotic carriers and assessing their ability to protect probiotics during the encapsulation process, storage, and in vitro simulation of gastrointestinal fluids. However, the carriers should help to deliver probiotics to the colon site to colonize and function. Consequently, it is essential to investigate more effective methods for developing colonic probiotic delivery systems and systematically investigate the related mechanisms referring to the relationship between carrier structure, cell viability, delivery efficiency, and release kinetics of probiotics.

Despite the successful preparation of probiotic formulations by electrohydrodynamic techniques, effective evaluation methods are also crucial. To date, most research has only examined the protective ability of the carriers employing in vitro simulated gastric fluid and/or intestinal fluid. These methods, however, cannot accurately reflect the actual condition of the gastrointestinal tract. Dynamic gastrointestinal models enriched with different digestion factors should be developed to provide valuable information on the delivery and survival of the encapsulated probiotics. Furthermore, in vivo tests should be designed and employed to examine the real effectiveness of formulations on probiotic stabilization and delivery. Even though many probiotics have been feasibly encapsulated, the final application of these items must be conducted by adding them to different foods. Regarding this, the survival of encapsulated probiotics in simulated or real food media should be investigated. Meanwhile, research efforts are still needed to ensure that probiotic-enriched foods do not undergo physicochemical sensory changes and that the minimum number of probiotics is maintained over shelf life and consumption.

Overall, electrospinning and electrospraying open new routes for stabilizing probiotics under different harsh conditions and provide new insight into a highly efficient usage of probiotics in various fields. In the future, more sophisticated preparation protocols based on electrohydrodynamic techniques and the exploration of suitable materials or their combinations are required to fabricate more efficient delivery vehicles for probiotics. Furthermore, a persistent effort should be made in promoting the application of electrohydrodynamic probiotic formulations in the food and pharmaceutical industries.

## Figures and Tables

**Table 1:** Overview of probiotic encapsulation by electrospinning using binary polymer systems.

Probiotic	Polymer	Additive	Diameter (nm)	Key Point	Ref.
*L. acidophilus*	Soluble dietary fiber (SDF), poly(vinyl alcohol) (PVA)	-	229–703	The addition of SDF improved the melting temperature of nanofibers, suggesting possible protection of probiotics in thermally processed foods.	[50]
*L. acidophilus*	PVA and polyvinylpyrrolidone (PVP)	-	142–934	Probiotic cells encapsulated in the nanofibers exhibited long term stability when stored under the temperature below 7 °C.	[75]
*L. rhamnosus*	Pectin (PEC), pullulan (PUL)	-	-	The development of PEC/PUL fiber mat excludes the usage of synthetic PEO and organic solvents, and is therefore promising in the food industry.	[73]
*L. plantarum*	PVA	Fructo-oligosaccharide (FOS)	410 ± 150	The addition of FOS during electrospinning improved the viability and thermal stability of *L. plantarum*.	[49]
*L. rhamnosus*	PVA, sodium alginate (SA)	-	60–580	The probiotic cells’ loaded nanofiber would be a promising coating material for fish fillets to prevent the rapid proliferation of total bacteria.	[76]
*L. brevis*, *L. reuteri*, *L. rhamnosus*	PVA, SA	-	-	The encapsulation within the SA/PVA fiber mat could prevent the pepsin-induced degradation of the carriers in simulated gastric juice.	[77]
*L. rhamnosus*	PVA, SA	-	60.09–522.1	The *L. rhamnosus*-loaded PVA/SA nanofibers provided better stable in terms of fatty acids in fish fillets.	[78]
Strain 25.2.M	PEO, chitosan (CS)	-	105 ± 30	The viability of probiotics in the carriers was preserved after 12 months of storage at room temperature, and release could be controlled by selecting the polymers.	[79]
*Enterococcus mundtii*	PVA, PVP	Glycerol	318 ± 12	The shelf-life test supported that the survival of probiotics encapsulated in the scaffold was increased by 2.78 ± 0.10 log10 CFU compared to the bio-dispersion.	[80]
*L. plantarum*	PEO	Lyo-protectant (i.e., sucrose, trehalose)	492 ± 35	The viability was not vitally influenced by the voltage and relative humidity used during the electrospinning. The addition of lyo-protectant in the fibers is beneficial for the survival due to the interactions between the lyo-protectant and probiotic cells.	[53]
*B. animalis*	PVA, CS	Inulin	117.5–217.6	The survival of probiotics loaded in CS/PVA/Inulin fibers were significantly increased under simulated gastric and intestinal fluids.	[81]
*L. paracasei*	PVA, SA	-	305	The encapsulated probiotics exhibited enhanced tolerance to simulated gastric juice and improved viability/survival in kefir, respectively. Incorporation of probiotics in kefir has no obvious influence on the characteristic pseudoplastic flow behavior and viscoelastic nature.	[82]
*Saccharomycopsis fibuligera* (*S. fibuligera*)	Wheat bran fiber, exopolysaccharide, PVP	-	250–300	The survival of encapsulated probiotics increased in comparison to the free cells during the in vitro digestion. The encapsulated cells could maintain their viability during 56 days of storage at 4 °C.	[83]
*L. fermentum*	PVA, SA	Inulin (P95, GR and HPX)	200–400	The survival of cells especially encapsulated in the fiber mat containing inulin showed higher viability against SGF and SIF.	[72]
*E. coli*	PEO, SA	Polysorbate 80	167 ± 23	2.74 × 10^5^ CFU/g of viable *E. coli* could be encapsulated in the fibers formed from the electrospun solution of 2.5/1.5/3 wt% SA/PEO/PS80.	[64]
*L. paracasei*	PVA, PEO	Glucose, lactose, mannitol, saccharose, trehalose, inulin, and skim milk	856–969	The use of excipients could reduce osmotic and dehydration stress during electrospinning and long-term storage, while increasing the survival of encapsulated cells.	[84]
*L. plantarum*	PVA, silk fibroin	-	190 ± 70	The encapsulated probiotics exhibited increased survival rates, after being treated in SGF for 2 h.	[85]
*L. acidophilus*, *L. rhamnosus*, *B. bifdum*, *B. animalis*	Corn starch, SA	-	~797	81–100% of the initial population retained viability after treatment in SGF.	[62]
*L. rhamnosus*, *L. acidophilus*, *B. bifidum*, and *B. animalis*	Corn starch, SA	-	~797	The encapsulation of probiotics in the SA/starch nanofiber mats had higher protective effects compared to the encapsulation method with a single biopolymer.	[74]
*L. rhamnosus*, *L. acidophilus*, *L. plantarum* and *L. casei*	Gum Arabic (GA), PUL	-	-	The GA/PUL (20:80) fibers ensure higher cell survivability than freeze-drying samples, and the encapsulated cells maintained viability during 28 days of storage at 4 °C.	[65]
*L. rhamnosus*	PVA, PEC	-	-	The survival rate of encapsulated *L. rhamnosus* 1.0320 encapsulated in the PVA/PEC nanofibers was 84.63% after 21 days of storage at 4 °C.	[86]
*L*. *acidophilus*	PVA, GA	-	~617	Free cells lost their vitality, while encapsulated cells maintained a viability count above the recommended level (10^7^ CFU) under simulated gastrointestinal conditions.	[87]
*Bacillus strains*	PEO, SA	-	200–300	Probiotics loaded in the nanofibers maintained good viability during the electrospinning and 6 months of storage at room temperature. Spores could be rapidly released from the PEO nanofibers, while presence of SA in the nanofiber prolonged their release.	[88]
*L. paragasseri*	PEO, SA	inulin	300–600	The probiotic form used in the electrospun samples influenced the release amount.	[89]
*L. acidophilus, Limosilactobacillus reuteri, Lacticaseibacillus casei, Lacticaseibacillus rhamnosus*	Gelatin (GE), SA	-	423–429	GE/SA nanofiber is a good platform for protecting live bacteria, inhibiting the growth of pathogenic bacteria, and extending the shelf life of fresh carp fillets under refrigerated conditions.	[90]

Note: *L., Lactobacillus*; *B., Bifidobacterium*.

**Table 2:** Overview of probiotic encapsulation by dry electrospraying (DE) and wet electrospraying (WE).

Probiotic	Polymer	Nozzle	Type	Size (μm)	Voltage (kV)	Distance (cm)	Flow Rate (mL/h)	Ref.
*B. animalis*	Whey protein concentrate (WPS), PUL	uniaxial	DE	0.259–0.658	12–14	7	0.3	[51]
*L. plantarum*	WPC, resistant starch (RS)	uniaxial	DE	20–40	10–14	10	0.15	[112]
*L. plantarum*	Shell: gelatin, Core: WPC	coaxial	DE	0.6 ± 0.39	17	10	Shell: 0.15, Core: 0.05	[113]
*B. longum* subsp. *infantis*	WPC	uniaxial	DE	2.47 ± 1.15	-	-	-	[114]
*L. rhamnosus*	Whey protein isolate (WPI), inulin, gum	uniaxial	DE	0.359–0.596	14	7	0.7	[115]
*L. casei*	WPC, WPI	uniaxial	DE	3.09 ± 1.04	14	10	0.5	[52]
*L. acidophilus*	WPI, lactose	uniaxial	DE	0.435	6–12	10	1	[111]
*Leuconostoc lactis*	Soy protein isolate (SPI)	uniaxial	DE	4.11	10–15	10	0.4	[116]
*B. animalis* subsp. *lactis*	Shell: Ethyl cellulose, Core: maltodextrin	coaxial	DE	3.33 ± 1.18	35	10	Shell: 0.42, core: 0.21	[53]
*L. acidophilus*	SA	uniaxial	WE	315 ± 56	4–10	6	10	[117]
*L. plantarum*	SA, PEC	uniaxial	WE	111–116	24	15	2	[118]
*L. plantarum*	SA	uniaxial	WE	100–300	24	15	2	[119]
*L. plantarum*	SA, CS	uniaxial	WE	300–450	9.5	10	5	[120]
*L. plantarum* and *B. lactis*	SA, CS, inulin, RS	uniaxial	WE	710–1040	9.5	10	5	[121]
*L. plantarum*	SA, CS, RS	uniaxial	WE	30–1300	7–16	10	-	[122]
*L. plantarum*	SA, PEC, SPI	coaxial	WE	~1400	12	10	Shell: 7, core: 3	[20]

Note: *L.*, *Lactobacillus*; *B.*, *Bifidobacterium*; DE, dry electrospraying; WE, wet electrospraying.

## Data Availability

Not applicable.

## References

[1] Hill C., Guarner F., Reid G., Gibson G.R., Merenstein D.J., Pot B., Morelli L., Canani R.B., Flint H.J., Salminen S. (2014). Expert consensus document: The international scientific association for probiotics and prebiotics consensus statement on the scope and appropriate use of the term probiotic. Nat. Rev. Gastro. Hepat..

[2] Fang K.L., Jin X., Hong S.H. (2018). Probiotic *Escherichia coli* inhibits biofilm formation of pathogenic *E. coli* via extracellular activity of DegP. Sci. Rep..

[3] Sen S., Mansell T.J. (2020). Yeasts as probiotics: Mechanisms, outcomes, and future potential. Fungal Genet. Biol..

[4] Bommasamudram J., Kumar P., Kapur S., Sharma D., Devappa S. (2022). Development of thermotolerant *Lactobacilli* cultures with improved probiotic properties using adaptive laboratory evolution method. Probiotics Antimicro..

[5] Farhangfar A., Gandomi H., Basti A.A., Misaghi A., Noori N. (2021). Study of growth kinetic and gastrointestinal stability of acid-bile resistant *Lactobacillus plantarum* strains isolated from Siahmazgi traditional cheese. Vet. Res. Forum.

[6] Yao M.F., Xie J.J., Du H.J., Mcclements D.J., Xiao H., Li L.J. (2020). Progress in microencapsulation of probiotics: A review. Compr. Rev. Food Sci. Food Saf..

[7] Juodeikiene G., Zadeike D., Bartkiene E., Lėlė V., Bernatoniene J., Jakštas V. (2019). A new delivery system based on apple pomace–pectin gels to encourage the viability of antimicrobial strains. Food Sci. Technol. Int..

[8] Frakolaki G., Giannou V., Kekos D., Tzia C. (2020). A review of the microencapsulation techniques for the incorporation of probiotic bacteria in functional foods. Crit. Rev. Food. Sci..

[9] Rodrigues F.J., Cedran M.F., Bicas J.L., Sato H.H. (2020). Encapsulated probiotic cells: Relevant techniques, natural sources as encapsulating materials and food applications-a narrative review. Food Res. Int..

[10] Zare M., Dziemidowicz K., Williams G.R., Ramakrishna S. (2021). Encapsulation of pharmaceutical and nutraceutical active ingredients using electrospinning processes. Nanomaterials.

[11] Rostamabadi H., Assadpour E., Tabarestani H.S., Falsafi S.R., Jafari S.M. (2020). Electrospinning approach for nanoencapsulation of bioactive compounds; recent advances and innovations. Trends Food Sci. Tech..

[12] Coelho S.C., Estevinho B.N., Rocha F. (2021). Encapsulation in food industry with emerging electrohydrodynamic techniques: Electrospinning and electrospraying–A review. Food Chem..

[13] Wen P., Zong M.H., Linhardt R.J., Feng K., Wu H. (2017). Electrospinning: A novel nano-encapsulation approach for bioactive compounds. Trends Food Sci. Technol..

[14] Xue J., Wu T., Dai Y.Q., Xia Y.N. (2019). Electrospinning and electrospun nanofibers: Methods, materials, and applications. Chem. Rev..

[15] Lim L.T., Mendes A.C., Chronakis I.S. (2019). Electrospinning and electrospraying technologies for food applications. Adv. Food Nutr. Res..

[16] Jacobsen C., García-Moreno P.J., Mendes A.C., Mateiu R.V., Chronakis I.S. (2018). Use of electrohydrodynamic processing for encapsulation of sensitive bioactive compounds and applications in food. Annu. Rev. Food Sci. Technol..

[17] Rostami M., Yousefi M., Khezerlou A., Mohammadi M.A., Jafari S.M. (2019). Application of different biopolymers for nanoencapsulation of antioxidants via electrohydrodynamic processes. Food Hydrocoll..

[18] Moreira A., Lawson D., Onyekuru L., Dziemidowicz K., Angkawinitwong U., Costa P.F., Radasci N., Williams G.R. (2020). Protein encapsulation by electrospinning and electrospraying. J. Control. Release.

[19] Chui C.Y., Odeleye A., Nguyen L., Kasoju N., Soliman E., Ye H. (2018). Electrosprayed genipin cross-linked alginate-chitosan microcarriers for ex vivo expansion of mesenchymal stem cells. J. Biomed. Mater. Res. A.

[20] Huang R.M., Feng K., Li S.F., Zong M.H., Han S.Y. (2021). Enhanced survival of probiotics in the electrosprayed microcapsule by addition of fish oil. J. Food Eng..

[21] Suksamran T., Opanasopit P., Rojanarata T., Ngawhirunpat T., Ruktanonchai U.P. (2009). Biodegradable alginate microparticles developed by electrohydrodynamic spraying techniques for oral delivery of protein. J. Microencapsul..

[22] Ghayempour S., Mortazavi S.M. (2013). Fabrication of micro–nanocapsules by a new electrospraying method using coaxial jets and examination of effective parameters on their production. J. Electrostat..

[23] Yao Z.C., Jin L.J., Ahamad Z., Huang J., Chang M.W., Li J.S. (2017). Ganoderma lucidum polysaccharide loaded sodium alginate micro-particles prepared via electrospraying in controlled deposition environments. Int. J. Pharmaceut..

[24] Pankongadisak P., Ruktanonchai R.U., Supaphol P.O. (2014). Preparation and characterization of silver nanoparticles-loaded calcium alginate beads embedded in gelatin scaffolds. AAPS PharmSciTech.

[25] Gryshkov O., Pogozhykh D., Zernetsch H., Hofmann N., Mueller T., Glasmacher B. (2014). Process engineering of high voltage alginate encapsulation of mesenchymal stem cells. Mat. Sci. Eng. C.

[26] Azad A.K., Al-Mahmood SM A., Chatterjee B., Sulaiman WM A.W., Elsayed T.M., Doolaanea A.A. (2020). Encapsulation of black seed oil in alginate beads as a pH-sensitive carrier for intestine-targeted drug delivery: *In vitro*, in vivo and ex vivo Study. Pharmaceutics.

[27] Zhao D., Li J.S., Suen W., Chang M.W., Huang J. (2016). Preparation and characterization of *Ganoderma lucidum* spores-loaded alginate microspheres by electrospraying. Mat. Sci. Eng. C.

[28] Klokk T.I., Melvik J.E. (2002). Controlling the size of alginate gel beads by use of a high electrostatic potential. J. Microencapsul..

[29] Bae J., Cho G.Y., Bai S.J. (2020). Alginate Hydrogel Beads for Immobilizing Single Photosynthetic Cells. Int. J. Precis. Eng. Manuf..

[30] Qayyum A.S., Jain E., Kolar G., Kim Y., Sell S.A., Zustiak S.P. (2017). Design of electrohydrodynamic sprayed polyethylene glycol hydrogel microspheres for cell encapsulation. Biofabrication.

[31] Panahi A., Pishevar A.R., Tavakoli M.R. (2020). Experimental investigation of electrohydrodynamic modes in electrospraying of viscoelastic polymeric solutions. Phys. Fluids.

[32] Khorram S.M. (2008). Electro-spray of high viscous liquids for producing mono-sized spherical alginate beads. Particuology.

[33] Ma Y.T., Björnmalm M., Wise A.K., Cortez-Jugo C., Revalor E., Ju Y., Feeney O.M., Richardson R.T., Hanssen E., Shepherd R.K. (2018). Gel-mediated electrospray assembly of silica supraparticles for sustained drug delivery. ACS Appl. Mater. Interfaces.

[34] Celebioglu A., Uyar T. (2020). Fast-dissolving antioxidant curcumin/cyclodextrin inclusion complex electrospun nanofibrous webs. Food Chem..

[35] Wen P., Zhu D.H., Feng K., Liu F.J., Lou W.Y., Li N., Zong M.H., Wu H. (2016). Fabrication of electrospun polylactic acid nanofilm incorporating cinnamon essential oil/β-cyclodextrin inclusion complex for antimicrobial packaging. Food Chem..

[36] Wen P., Hu T.G., Li L., Zong M.H., Wu H. (2018). A colon-specific delivery system for quercetin with enhanced cancer prevention based on co-axial electrospinning. Food Funct..

[37] Yang H., Feng K., Wen P., Zong M.H., Lou W.Y., Wu H. (2017). Enhancing oxidative stability of encapsulated fish oil by incorporation of ferulic acid into electrospun zein mat. LWT-Food Sci. Technol..

[38] Feng K., Wen P., Yang H., Lou W.Y., Li N., Zong M.H., Wu H. (2017). Enhancement of the antimicrobial activity of cinnamon essential oil-based electrospun nanofilm by incorporation of lysozyme. RSC Adv..

[39] Feng K., Li S.H., Wei Y.S., Zong M.H., Hu T.G., Wu H., Han S.Y. (2021). Fabrication of nanostructured multi-unit vehicle for intestinal-specific delivery and controlled release of peptide. Nanotechnology.

[40] Li C., Wei Y.S., Wen P., Feng K., Zong M.H., Wu H. (2018). Preparation and characterization of an electrospun colon-specific delivery system for salmon calcitonin. RSC Adv..

[41] Padrão J.R., Casal M., Lanceros-Méndez S., Rodrigues L.R., Dourado F., Sencadas V. (2015). Antibacterial performance of bovine lactoferrin-fish gelatin electrospun membranes. Int. J. Biol. Macromol..

[42] Wen P., Hu T.G., Wen Y., Linhardt R.J., Zong M.H., Zou Y.X., Wu H. (2019). Targeted delivery of phycocyanin for the prevention of colon cancer using electrospun fibers. Food Funct..

[43] Feng K., Li C., Wei Y.S., Zong M.H., Wu H. (2019). Development of a polysaccharide based multi-unit nanofiber mat for colon-targeted sustained release of salmon calcitonin. J. Colloid Interface Sci..

[44] Wang C., Ma Chao Wu Z.K., He L., Yan P., Song J., Ma N., Zhao Q.H. (2015). Enhanced bioavailability and anticancer effect of curcumin-loaded electrospun nanofiber: In vitro and in vivo study. Nanoscale Res. Lett..

[45] Yang H., Wen P., Feng K., Zong M.H., Lou W.Y., Wu H. (2017). Encapsulation of fish oil in a coaxial electrospun nanofibrous mat and its properties. RSC Adv..

[46] Feng K., Zhai M.Y., Wei Y.S., Zong M.H., Wu H., Han S.Y. (2021). Fabrication of nano/micro-structured electrospun detection card for the detection of pesticide residues. Foods.

[47] Khan R.S., Rather A.H., Wani T.U., Rather S.U., Amna T., Hassan M.S., Sheikh F.A. (2023). Recent trends using natural polymeric nanofibers as supports for enzyme immobilization and catalysis. Biotechnol. Bioeng..

[48] Fung W.Y., Yuen K.H., Liong M.T. (2011). Agrowaste-based nanofibers as a probiotic encapsulant: Fabrication and characterization. J. Agr. Food Chem..

[49] Feng K., Huang R.M., Wu R.Q., Wei Y.S., Zong M.H., Linhardt R.J., Wu H. (2020). A novel route for double-layered encapsulation of probiotics with improved viability under adverse conditions. Food Chem..

[50] Škrlec K., Zupančič Š., Mihevc S.P., Kocbek P., Kristl J., Berlec A. (2019). Development of electrospun nanofibers that enable high loading and long-term viability of probiotics. Eur. J. Pharm. Biopharm..

[51] López-Rubio A., Sanchez E., Wilkanowicz S., Sanz Y., Lagaron J.M. (2012). Electrospinning as a useful technique for the encapsulation of living *Bifidobacteria* in Food hydrocolloid. Food Hydrocoll..

[52] Alehosseini A., Sarabi-Jamab M., Ghorani B., Kadkhodaee R. (2019). Electro-encapsulation of Lactobacillus casei in high-resistant capsules of whey protein containing transglutaminase enzyme. LWT-Food Sci. Technol..

[53] Moreno J.S., Dima P., Chronakis I.S., Mendes A.C. (2022). Electrosprayed ethyl cellulose core-shell microcapsules for the encapsulation of probiotics. Pharmaceutics.

[54] Lee S.W., Belcher A.M. (2004). Virus-based fabrication of micro- and nanofibers using electrospinning. Nano Lett..

[55] Salalha W., Kuhn J., Dror Y., Zussman E. (2006). Encapsulation of bacteria and viruses in electrospun nanofibres. Nanotechnology.

[56] Heunis T.D., Botes J.M., Dicks LM T. (2010). Encapsulation of *Lactobacillus plantarum* 423 and its bacteriocin in nanofibers. Probiotics Antimicrob. Proteins.

[57] Gensheimer M., Becker M., Brandis-Heep A., Wendorff J.H.,  Thauer R.K., Greiner A. (2007). Novel biohybrid materials by electrospinning: Nanofibers of poly(ethylene oxide) and living bacteria. Adv. Mater..

[58] Zupančič Š., Škrlec K., Kocbek P., Kristl J., Berlec A. (2019). Effects of electrospinning on the viability of ten species of Lactic acid bacteria in poly(ethylene oxide) nanofibers. Pharmaceutics.

[59] Krasowska A., Sigler K. (2014). How microorganisms use hydrophobicity and what does this mean for human needs?. Front. Cell. Infect. Microbiol..

[60] Feng K., Zhai M.Y., Zhang Y., Linhardt R.J., Zong M.H., Li L., Wu H. (2018). Improved viability and thermal stability of the probiotics encapsulated in a novel electrospun fiber mat. J. Agr. Food Chem..

[61] Ceylan Z., Uslu E., Spirli H., Meral R., Gavgali M., Yilmaz M.T., Dertli E. (2019). A novel perspective for *Lactobacillus reuteri*: Nanoencapsulation to obtain functional fish fillets. LWT-Food Sci. Technol..

[62] Atraki R., Azizkhani M. (2021). Survival of probiotic bacteria nanoencapsulated within biopolymers in a simulated gastrointestinal model. Innov. Food Sci. Emerg. Technol..

[63] Hajikhani M., Lin M.S. (2022). A review on designing nanofibers with high porous and rough surface via electrospinning technology for rapid detection of food quality and safety attributes. Trends Food Sci. Technol..

[64] Diep E., Schiffman J.D. (2021). Encapsulating bacteria in alginate-based electrospun nanofibers. Biomater. Sci..

[65] Ma J.G., Xu C., Yu H.L., Feng Z.B., Yu W., Gu L.Y., Liu Z.J., Chen L.J., Jiang Z.M., Hou J.C. (2021). Electro-encapsulation of probiotics in gum Arabic-pullulan blend nanofibres using electrospinning technology. Food Hydrocoll..

[66] Stojanov S., Plavec T.V., Kristl J., Zupančič Š., Berlec A. (2021). Engineering of vaginal Lactobacilli to express fluorescent proteins enables the analysis of their mixture in nanofibers. Int. J. Mol. Sci..

[67] Kumar K., Zare E.N., Torres-Mendieta R., Wacawek S., Makvandi P., Černík M., Padil VV T., Varma R.S. (2021). Electrospun fibers based on botanical, seaweed, microbial, and animal sourced Biomacromolecules and their multidimensional applications. Int. J. Biol. Macromol..

[68] Kumar T.S.M., Kumar K.S., Rajini N., Siengchin S., Ayrilmis N., Rajulu A.V. (2019). A comprehensive review of electrospun nanofibers: Food and packaging perspective. Compos. Part B Eng..

[69] Salminen S., Collado M.C., Endo A., Hill C., Lebeer S., Quigley E.M., Sanders M.E., Shamir R., Swann J.R.M., Szajewska H. (2021). The International Scientific Association of Probiotics and Prebiotics (ISAPP) consensus statement on the definition and scope of postbiotics. Nat. Rev. Gastroenterol. Hepatol..

[70] Ta L.P., Bujna E., Antal O., Ladányi M., Juhász R., Szécsi A., Kun S., Sudheer S., Gupta V.K., Nguyen Q.D. (2021). Effects of various polysaccharides (alginate, carrageenan, gums, chitosan) and their combination with prebiotic saccharides (resistant starch, lactosucrose, lactulose) on the encapsulation of probiotic bacteria *Lactobacillus casei* 01 strain. Int. J. Biol. Macromol..

[71] Luca L., Oroian M. (2021). Influence of different prebiotics on viability of *Lactobacillus casei*, *Lactobacillus plantarum* and *Lactobacillus rhamnosus* encapsulated in alginate microcapsules. Foods.

[72] Duman D., Karadag A. (2021). Inulin added electrospun composite nanofibres by electrospinning for the encapsulation of probiotics: Characterisation and assessment of viability during storage and simulated gastrointestinal digestion. Int. J. Food Sci. Technol..

[73] Liu S.C., Li R., Tomasula P.M., Sousa AM M., Liu L.S. (2016). Electrospun food-grade ultrafine fibers from pectin and pullulan blends. Food Nutr. Sci..

[74] Ghorbani S., Maryam A. (2021). Encapsulation of *lactic acid bacteria* and *Bifidobacteria* using starch-sodium alginate nanofibers to enhance viability in food model. J. Food Process. Pres..

[75] Nagy Z.K., Wagner I., Suhajda Á., Tobak T., Harasztos A.H., Vigh T., Sóti P.L., Pataki H., Molnár K., Marosi G. (2014). Nanofibrous solid dosage form of living bacteria prepared by electrospinning. Express Polym. Lett..

[76] Ceylan Z., Meral R., Karakas C.Y., Dertli E., Yilmaz M.T. (2018). A novel strategy for probiotic bacteria: Ensuring microbial stability of fish fillets using characterized probiotic bacteria-loaded nanofibers. Innov. Food Sci. Emerg. Technol..

[77] Karakas C.K., Duman D., Yilmaz M.T. (2018). Encapsulation of probiotic living cells in alginate-PVA based electrospun nanofibers: Evaluation of viability and survival in simulated gastrointestinal conditions. J. Biotechnol..

[78] Ceylan Z., Meral R., Cavidoglu I., Karakas C.Y., Yilmaz M.T. (2018). A new application on fatty acid stability of fish fillets: Coating with probiotic bacteria-loaded polymer-based characterized nanofibers. J. Food Saf..

[79] Zupančič Š., Rijavec T., Lapanje A., Petelin M., Kristl J., Kocbek P. (2018). Nanofibers with incorporated autochthonous bacteria as potential probiotics for local treatment of periodontal disease. Biomacromolecules.

[80] Khan M.A., Hussain Z., Ali S., Qamar Z., Imran M., Hafeez Y. (2019). Fabrication of electrospun probiotic functionalized nanocomposite scaffolds for infection control and dermal burn healing in a mice model. ACS Biomater. Sci. Eng..

[81] Mojaveri S.J., Hosseini S.F., Gharsallaoui A. (2020). Viability improvement of *Bifidobacterium animalis* bb12 by encapsulation in chitosan/poly(vinyl alcohol) hybrid electrospun fiber mats. Carbohydr. Polym..

[82] Yilmaz M.T., Taylan O., Karakas C.Y., Dertli E. (2020). An alternative way to encapsulate probiotics within electrospun alginate nanofibers as monitored under simulated gastrointestinal conditions and in kefir. Carbohydr. Polym..

[83] Ragavan M.L., Das N. (2020). Nanoencapsulation of *Saccharomycopsis fibuligera* VIT-MN04 using electrospinning technique for easy gastrointestinal transit. IET Nanobiotechnol..

[84] Hirsch E., Pantea E., Vass P., Domján J., Molnár M., Suhajda Á., Andersen S.K., Vigh T., Verreck M., Marosi G. (2021). Probiotic bacteria stabilized in orally dissolving nanofibers prepared by high-speed electrospinning. Food Bioprod. Process..

[85] Wei L.L., Zhou D., Kang X.J. (2021). Electrospinning as a novel strategy for the encapsulation of living probiotics in polyvinyl alcohol/silk fibroin. Innov. Food Sci. Emerg. Technol..

[86] Xu C., Ma J., Wang W., Liu Z.J., Gu L.Y., Qian S.S., Hou J.C. (2022). Preparation of pectin-based nanofibers encapsulating *Lactobacillus rhamnosus* 1.0320 by electrospinning. Food Hydrocoll..

[87] Fareed F., Saeed F., Afzaal M., Imran A., Ahmad A., Mahmood K., Shah Y.A., Hussain M., Ateeq H. (2022). Fabrication of electrospun gum Arabic–polyvinyl alcohol blend nanofibers for improved viability of the probiotic. J. Food Sci. Technol..

[88] Grilc N.K., Zidar A., Kocbek P., Rijavec T., Colja T., Lapanje A., Jeras M., Gobec M., Mlinarič-Raščan I., Gašperlin M. (2023). Nanofibers with genotyped *Bacillus* strains exhibiting antibacterial and immunomodulatory activity. J. Control. Release.

[89] Simonič M., Slapničar Š., Trček J., Matijašić B.B., Lorbeg P.M., Vesel A., Zemljič L.F., Fratnik Z.P. (2023). Probiotic *Lactobacillus paragasseri* K7 nanofiber encapsulation using nozzle-free electrospinning. Appl. Biochem. Biotechnol..

[90] Ghalehjooghi H.D., Tajik H., Shahbazi Y. (2023). Development and characterization of active packaging nanofiber mats based on gelatin-sodium alginate containing probiotic microorganisms to improve the shelf-life and safety quality of silver carp fillets. Int. J. Food Microbiol..

[91] López-Rubio A., Sanchez E., Sanz Y., Lagaron J.M. (2009). Encapsulation of living Bifidobacteria in ultrathin PVOH electrospun fibers. Biomacromolecules.

[92] Lancuški A., Ammar A.A., Avrahami R., Vilensky R., Vasilyev G., Zussman E. (2017). Design of starch-formate compound fibers as encapsulation platform for biotherapeutics. Carbohydr. Polym..

[93] Feng K., Wei Y.S., Hu T.G., Linhardt R.J., Zong M.H., Wu H. (2020). Colon-targeted delivery systems for nutraceuticals: A review of current vehicles, evaluation methods and future prospects. Trends Food Sci. Technol..

[94] Cui M.X., Zhang M., Liu K.H. (2021). Colon-targeted drug delivery of polysaccharide-based nanocarriers for the synergistic treatment of inflammatory bowel disease:A review. Carbohydr. Polym..

[95] Ribeiro J.A., Pereira E.D., Raphaelli C.D., Radünz M., Camargo T.M., Cocenco F.I.G.D., Radunz M., Camargo T.M., Cantillano R.F.F., Fiorentini Â.M. (2021). Application of prebiotics in apple products and potential health benefits. J. Food Sci. Technol..

[96] Yu H.L., Liu W.H., Li D.M., Liu C.C., Feng Z.B., Jiang B. (2020). Targeting delivery system for Lactobacillus plantarum based on functionalized electrospun nanofibers. Polymers.

[97] Xu C., Ma J.G., Liu Z.J., Wang W., Liu X., Qian S.S., Chen L.J., Gu L.Y., Sun C.Q., Hou J.C. (2023). Preparation of shell-core fiber-encapsulated *Lactobacillus rhamnosus* 1.0320 using coaxial electrospinning. Food Chem..

[98] Çanga E.M., Dudak F.C. (2021). Improved digestive stability of probiotics encapsulated within poly(vinyl alcohol)/cellulose acetate hybrid fibers. Carbohydr. Polym..

[99] Ajalloueian F., Guerra P.R., Bahl M.I., Trop A.M., Hwu E.T., Licht T.R., Boisen A. (2022). Multi-layer PLGA-pullulan-PLGA electrospun nanofibers for probiotic delivery. Food Hydrocoll..

[100] Jun I., Han H.S., Edwards J.R., Jeon H. (2018). Electrospun fibrous scaffolds for tissue engineering: Viewpoints on architecture and fabrication. Int. J. Mol. Sci..

[101] San N.O., Celebioglu A., Tümtaş Y., Uyar T., Tekinay T. (2014). Reusable bacteria immobilized electrospun nanofibrous webs for decolorization of methylene blue dye in wastewater treatment. RSC Adv..

[102] Sarioglu O.F., Keskin N., Celebioglu A., Tekinay T., Uyar T. (2017). Bacteria immobilized electrospun polycaprolactone and polylactic acid fibrous webs for remediation of textile dyes in water. Chemosphere.

[103] Hu M.X., Li J.N., Guo Q., Zhu Y.Q., Niu H.M. (2019). Probiotics biofilm-integrated electrospun nanofiber membranes: A new starter culture for fermented milk production. J. Agr. Food Chem..

[104] Valamehr B., Jonas S.J., Polleux J., Qiao R., Guo S., Gschweng E.H., Stiles B., Kam K., Luo T.J., Witte O.N. (2008). Hydrophobic surfaces for enhanced differentiation of embryonic stem cell-derived embryoid bodies. Proc. Natl. Acad. Sci. USA.

[105] Anselme K., Davidson P., Popa A.M., Giazzon M., Liley M., Ploux L. (2010). The interaction of cells and bacteria with surfaces structured at the nanometre scale. Acta Biomater..

[106] Shi J., Li S.F., Feng K., Han S.Y., Hu T.G., Wu H. (2022). Improving the viability of probiotics under harsh conditions by the formation of biofilm on electrospun nanofiber mat. Foods.

[107] Berezina O.Y., Vasilyeva A.V., Sidorova N.A., Savushkin A.I., Marcova N.P. (2019). The effect of polyvinylpyrrolidone nanowires on the metabolic activity of *Lactobacillus acidophilus*. IOP Conf. Ser. Mater. Sci. Eng..

[108] Jayani T., Sanjeev B., Marimuthu S., Uthandi S. (2020). Bacterial cellulose nano fiber (BCNF) as carrier support for the immobilization of probiotic, *Lactobacillus acidophilus* 016. Carbohydr. Polym..

[109] Grzywaczyk A., Zdarta A., Jankowska K., Biadasz A., Zdarta J., Jesionowski T., Kaczorek E., Smułek W. (2021). New biocomposite electrospun fiber/alginate hydrogel for probiotic bacteria immobilization. Materials.

[110] Harandi F.N., Khorasani A.C., Shojaosadati S.A., Hashemi-Najafabadi S. (2022). Surface modification of electrospun wound dressing material by Fe_2_O_3_ nanoparticles incorporating Lactobacillus strains for enhanced antimicrobial and antibiofilm activity. Surf. Interfaces.

[111] Amiri S., Teymorlouei M.J., Bari M.R., Khaledabad M.A. (2021). Development of *Lactobacillus acidophilus* LA5-loaded whey protein isolate/lactose bionanocomposite powder by electrospraying: A strategy for entrapment. Food Biosci..

[112] Gómez-Mascaraque L.G., Morfin R.C., Pérez-Masiá R., Sanchez G., López-Rubio A. (2016). Optimization of electrospraying conditions for the microencapsulation of probiotics and evaluation of their resistance during storage and *in-vitro* digestion. LWT-Food Sci. Technol..

[113] Gómez-Mascaraque L.G., Ambrosio-Martín J., Perez-Masiá R., López-Rubio A. (2017). Impact of acetic acid on the survival of *L. plantarum* upon microencapsulation by coaxial electrospraying. J. Healthc. Eng..

[114] Librán C.M., Castro S., Lagaron J.M. (2017). Encapsulation by electrospray coating atomization of probiotic strains. Innov. Food. Sci. Emerg. Technol..

[115] Moayyedi M., Eskandari M.H., Rad AH E., Ziaee E., Khodaparast MH H., Golmakani M.T. (2018). Effect of drying methods (electrospraying, freeze drying and spray drying) on survival and viability of microencapsulated *Lactobacillus rhamnosus* ATCC 7469. J. Funct. Foods.

[116] Premjit Y., Mitra J. (2021). Optimization of electrospray-assisted microencapsulation of probiotics (*Leuconostoc lactis*) in soy protein isolate-oil particles using Box-Behnken experimental design. Food Bioprocess Technol..

[117] Laelorspoen N., Wongsasulak S., Yoovidhya T., Devahastin S. (2014). Microencapsulation of *lactobacillus acidophilus* in zein-alginate core-shell microcapsules via electrospraying. J. Funct. Foods.

[118] Coghetto C.C., Brinques G.B., Siqueira N.M., Pletsch J., Soares RM D., Ayub MA Z. (2016). Electrospraying microencapsulation of *Lactobacillus plantarum* enhances cell viability under refrigeration storage and simulated gastric and intestinal fluids. J. Funct. Foods.

[119] Coghetto C.C., Flores S.H., Brinques G.B., Ayub MA Z. (2016). Viability and alternative uses of a dried powder, microencapsulated *Lactobacillus plantarum* without the use of cold chain or dairy products. LWT-Food Sci. Technol..

[120] Zaeim D., Sarabi-Jamab M., Ghorani B., Kadkhoda R., Tromp R.H. (2017). Electrospray assisted fabrication of hydrogel microcapsules by single-and double-stage procedures for encapsulation of probiotics. Food Bioprod. Process..

[121] Zaeim D., Sarabi-Jamab M., Ghorani B., Kadkhodaee R. (2019). Double layer co-encapsulation of probiotics and prebiotics by electro-hydrodynamic atomization. LWT-Food Sci. Technol..

[122] Ta L.P., Bujna E., Kun S., Charalampopoulos D., Khutoryanskiy V.V. (2021). Electrosprayed mucoadhesive alginate-chitosan microcapsules for gastrointestinal delivery of probiotics. Int. J. Pharmaceut..

[123] Fritzen-Freire C.B., Prudencio E.S., Amboni RD M.C., Pinto S.S., Negrao-Murakami A.N., Murakami F.S. (2012). Microencapsulation of Bifidobacteria by spray drying in the presence of prebiotics. Food Res. Int..

[124] Raddatz G.C., Poletto G., Deus C.D., Codevilla C.F., Menezes C. (2019). Use of prebiotic sources to increase probiotic viability in pectin microparticles obtained by emulsification/internal gelation followed by freeze-drying. Food Res. Int..

[125] Gandomi H., Abbaszadeh S., Misaghi A., Bokaie S., Noori N. (2016). Effect of chitosan-alginate encapsulation with inulin on survival of *Lactobacillus rhamnosus GG* during apple juice storage and under simulated gastrointestinal conditions. LWT-Food Sci. Technol..

[126] Gibson G.R., Hutkins R., Sanders M.E., Prescott S.L., Reimer R.A., Salminen S.J., Scott K., Stanton C., Swanson K.S., Cani P.D. (2017). The International Scientific Association for Probiotics and Prebiotics (ISAPP) consensus statement on the definition and scope of prebiotics. Nat. Rev. Gastroenterol. Hepatol..

[127] Eratte D., Dowling K., Barrow C.J., Adhikari B.P. (2017). *In-vitro* digestion of probiotic bacteria and omega-3 oil co-microencapsulated in whey protein isolate-gum Arabic complex coacervates. Food Chem..

[128] Harandi F.N., Khorasani A.C., Shojaosadati S.A., Hashemi-Najafabadi S. (2021). Living Lactobacillus-ZnO nanoparticles hybrids as antimicrobial and antibiofilm coatings for wound dressing application. Mat. Sci. Eng. C-Mater..

[129] Iglesias M.B., Echeverría G., Viñas I., López M.L., Abadias M. (2018). Biopreservation of fresh-cut pear using *Lactobacillus rhamnosus GG* and effect on quality and volatile compounds. LWT-Food Sci. Technol..

